# FGF11 influences 3T3‐L1 preadipocyte differentiation by modulating the expression of PPARγ regulators

**DOI:** 10.1002/2211-5463.12619

**Published:** 2019-03-12

**Authors:** Kyeong Won Lee, Jae‐Yeon Jeong, Young Jun An, Jung‐Hyun Lee, Hyung‐Soon Yim

**Affiliations:** ^1^ Marine Biotechnology Research Center Korea Institute of Ocean Science and Technology Busan Korea; ^2^ Department of Marine Biotechnology Korea University of Science and Technology Daejeon Korea

**Keywords:** 3T3‐L1, adipocyte, adipogenesis, fibroblast growth factor 11, PPARγ

## Abstract

Fibroblast growth factor 11 (FGF11) is a member of the intracellular fibroblast growth factor superfamily. Here, we identified FGF11 as a novel mediator of adipogenesis. During 3T3‐L1 adipocyte differentiation, the expression of FGF11 decreased at the mitotic clonal expansion stage and increased at the terminal differentiation stage. FGF11 knockdown reduced the expression of peroxisome proliferator‐activated receptor gamma (PPARγ), a master regulator of adipogenesis, resulting in the inhibition of adipocyte differentiation. Treatment with the PPARγ agonist rosiglitazone restored the inhibition of adipogenesis caused by FGF11 knockdown. We also report that the expression of the PPARγ regulators CCAAT/enhancer‐binding protein α, sterol regulatory element‐binding protein 1, KLF9, KLF2, GATA binding factor 2, and GATA binding factor 3 was influenced by FGF11. These results suggest that FGF11 indirectly controls the expression of PPARγ through modifying the expression of multiple PPARγ regulators, thereby mediating adipogenesis.

**Abbreviations**
C/EBP: CCAAT/enhancer‐binding protein
FGF: fibroblast growth factor
GATA2: GATA binding factor 2
GATA3: GATA binding factor 3
iFGF: intracellular fibroblast growth factor
KLF: Krüppel‐like factor
MCE: mitotic clonal expansion
NFI: nuclear factor I
NLS: nuclear localization sequence
PPAR: peroxisome proliferator‐activated receptor
SREBP1: sterol regulatory element‐binding protein 1
ZFP423: zinc finger protein 423

The family of fibroblast growth factors (FGFs) is composed of 22 members and is associated with various biological functions, such as growth, wound healing, repair, differentiation, angiogenesis, embryonic development, and metabolic regulation 1. FGFs can be classified into canonical FGFs, endocrine FGFs, and intracellular FGFs (iFGFs). Canonical and endocrine FGFs function by binding to FGF receptors (FGFRs) with heparin sulfate proteoglycans or klotho proteins, whereas the iFGFs FGF11‐FGF14 act as intracellular molecules in an FGFR‐independent manner 2. iFGFs are mainly expressed in the nervous system, and much of the research concerning them has focused on neuronal development 3. In contrast to other iFGFs, the function and molecular mechanism of FGF11 activity have not been studied well. Recently, our group reported that the promoter of FGF11 contains a hypoxia response element (HRE), and FGF11 expression is induced under hypoxic conditions 4. We also identified FGF11 as a stabilizer of HIF‐1α and an enhancer of capillary‐like endothelial tube formation 5, 6. It was also documented that FGF11 is related to osteoclast‐mediated bone resorption, tumorigenesis, and liver regeneration 7. Transcriptome analysis of human adipose‐derived stem cells during adipogenesis suggested that FGF11 is a potential mediator of adipogenesis 8; however, further study is needed to determine the detailed functions and regulatory mechanisms of FGF11 in this process.

Adipocyte differentiation is an orchestrated process controlled by a cascade of multiple regulators. The 3T3‐L1 cell line is a well‐established model for studying adipogenesis 9. Adipocyte differentiation can be divided into two phases: the mitotic clonal expansion (MCE) phase and the terminal differentiation phase 10. Upon treatment with adipogenic inducers (dexamethasone, IBMX, and insulin, also called DMI), growth‐arrested 3T3‐L1 preadipocytes re‐enter the cell cycle and then undergo 2–3 rounds of mitosis 11. After entering MCE, the adipogenic genes are expressed according to a precise temporal process, ultimately leading to the terminal differentiation phase. CCAAT/enhancer‐binding protein β (C/EBPβ), which is temporally increased in the MCE phase, has been identified as an important regulator that promotes MCE and initiates terminal differentiation. In terminal adipocyte differentiation, peroxisome proliferator‐activated receptor gamma (PPARγ) and CCAAT/enhancer‐binding protein α (C/EBPα), essential adipogenic regulators, are induced by C/EBPβ. PPARγ and C/EBPα positively regulate each other, and the cooperation of these two proteins drives the expression of genes involved in the adipogenic phenotype, such as morphological changes, lipid accumulation, and insulin sensitivity 12.

Understanding the complexity of adipocyte differentiation is of important relevance to human health, because adipocyte dysfunction contributes to metabolic diseases. In the present study, we found that the expression of FGF11 changed dramatically between the two phases of adipogenesis, and the increase of FGF11 in the terminal differentiation phase was necessary for adipocyte maturation. The expression of PPARγ contributed to the effect of FGF11 on adipogenesis, and FGF11 altered the expression of a regulator of PPARγ expression. This study provides insight into a novel role for FGF11 in adipogenesis.

## Materials and methods

### Preparation of adenoviruses and siRNAs

Adenoviruses encoding FLAG‐tagged human FGF11 (Ad‐FLAG‐FGF11) and control adenoviruses (Ad‐GFP) were prepared as previously described. Briefly, FLAG‐FGF11 cDNA was inserted into the pAdTrack‐CMV‐expressing GFP vector followed by homologous recombination with pAdEasy‐1, an adenoviral backbone vector (Agilent Technologies, Palo Alto, CA, USA). The Ad‐FLAG‐FGF11 adenoviruses were generated by transfection of a linearized pAd‐FLAG‐FGF11 into AD‐293 cells (Agilent Technologies). The siRNAs against mouse FGF11 (Stealth siRNAs #MSS247199) and the negative control siRNAs (siNS) (AccuTarget™ Negative Control siRNA #SN‐1013) were purchased from Invitrogen (Carlsbad, CA, USA) and Bioneer (Daejeon, Korea), respectively.

### Cell culture

3T3‐L1 preadipocytes were cultured in Dulbecco's modified Eagle's medium (DMEM) supplemented with 10% bovine serum and 1% penicillin/streptomycin in a 5% CO_2_ incubator (GIBCO, Life Technologies Ltd., Paisley, UK). Two days after the cells had reached confluence, differentiation was induced. Cells were incubated in culture media with DMEM containing 10% FBS, 0.5 μm 3‐isobutyl‐1‐methylxanthine (IBMX), 0.25 μm dexamethasone, and 5 μg·mL^−1^ insulin for 2 days. Then, cells were incubated in DMEM with 10% FBS and 1 μg·mL^−1^ insulin for the following 2 days and were then further cultured in DMEM with 10% FBS for another 4 days. For assessment of adipocyte differentiation, after fixation with 3.5% formaldehyde and washing with PBS, lipid droplets were stained with 1.5% Oil Red O (ORO) in 60% isopropanol for 1 h and washed with PBS to remove the unbound stain. To quantify the ORO content, DMSO was added to each sample, and the absorbance of each sample was measured at 510 nm.

### Transient transfection of siRNAs and transduction with adenoviruses

For FGF11 knockdown, cells were transfected with siRNAs (50 nm) using Lipofectamine RNAiMAX (Invitrogen) 1 day before the induction of adipogenesis. The next day, the culture medium was replaced with differentiation medium. FGF11 was overexpressed by poly‐l‐lysine (PLL; P9155; Sigma‐Aldrich, St. Louis, MO, USA)‐assisted transduction 2 days after the induction of adipogenesis. Briefly, adenoviruses and PLL (2.5 μg·mL^−1^) were incubated in serum‐free medium for 30 min. Then, cells were incubated in this mixture for 1 h, followed by the addition of insulin‐supplemented medium. After 1 day, the culture medium was replaced with fresh insulin‐supplemented medium.

### Western blot analysis

To assess the protein levels, cell lysates were prepared using lysis buffer containing 20 mm Tris/HCl (pH 7.4), 1% NP‐40, 5 mm EDTA, 100 mm NaF, 2 mm Na_3_VO_4_, 10 mm Na_4_P_2_O_7_, and protease inhibitor cocktail (#862209; ThermoFisher Scientific, Waltham, MA, USA). Whole‐cell lysates (10 μg) were subjected to SDS/PAGE and immunoblotted with specific antibodies. For immunoblotting, anti‐PPARγ (sc‐7273; Santa Cruz Biotech, Santa Cruz, CA, USA), anti‐C/EBPβ (sc‐7962; Santa Cruz Biotech), C/EBPα (sc‐61; Santa Cruz Biotech), anti‐β‐catenin (#610154; BD Transduction Laboratories, San Jose, CA, USA), anti‐γ‐tubulin (T6557; Sigma‐Aldrich), anti‐FLAG (F1804; Sigma‐Aldrich), phospho‐Y654 FGFR1 (ab59194; Abcam, Cambridge, MA, USA), FGFR1 (#9740; Cell Signaling Technology, Beverly, MA, USA), phospho Y653/Y654 FGFR1‐4 (AF3285; R&D Systems, Minneapolis, MN, USA), FGFR2 (ab109372; Abcam), FGFR3 (ab133644; Abcam), and FGFR4 (ab119378; Abcam) antibodies were used. The proteins were visualized using Clarity™ Western ECL Blotting Substrates (Bio‐Rad, Hercules, CA, USA) or ECL‐Prime (ThermoFisher Scientific) and ChemiDoc Imaging System (Bio‐Rad).

### RNA preparation and real‐time RT/PCR

Total RNA was extracted using TRIzol (Invitrogen) according to the manufacturer's instructions, and cDNA was synthesized using MMLV reverse transcriptase and random hexamers (Invitrogen) with 1 μg of total RNA. The levels of each gene transcript were analyzed by real‐time PCR with gene‐specific primers (Table 1), and 18s rRNA was used as an endogenous control. Real‐time PCR was performed using Thunderbird SYBR qPCR Mix (TOYOBO, Osaka, Japan) and a StepOnePlus Real‐Time PCR System (Applied Biosystems, Foster City, CA, USA).

**Table 1:** Primers used for real‐time RT/PCR

Primers	Sequence	Species
FGF11	F 5′‐GCCAAGCTGGGTCACTACAT‐3′	Mouse
	R 5′‐GCTGCCTTGGTCTTCTTGAC‐3′	
FGF11 isoform 1	F 5′‐GCCAAGCTGGGTCACTACAT‐3′	
	R 5′‐ACGCACTCCTTAAAGCGACA‐3′	
C/EBPβ	F 5′‐AAGGCCAAGGCCAAGAAGA‐3′	
	R 5′‐TTGTGCTGCGTCTCCAGG‐3′	
PPARγ	F 5′‐TCATGACCAGGGAGTTCCTC‐3′	
	R 5′‐GGCGGTCTCCACTGAGAATA‐3′	
C/EBPα	F 5′‐AGTCGGTGGACAAGAACAGC‐3′	
	R 5′‐GTCACTGGTCAACTCCAGCA‐3′	
KLF9	F 5′‐CACACTGGGGAAAAGCAGTT‐3′	
	R 5′‐ATCATGCTGGGATGGAACTC‐3′	
ZFP423	F 5′‐AAACACAAGAGGAGCCGAGA‐3′	
	R 5′‐CTGTGGGTCTTCAGGTGGAT‐3′	
NF1	F 5′‐CTGGAGAGCACAGATGGTGA‐3′	
	R 5′‐GTAGGCCAGGTACAGGTCCA‐3′	
SREBP1	F 5′‐TACTTCTTGTGGCCCGTACC‐3′	
	R 5′‐TCAGGTCATGTTGGAAACCA‐3′	
GATA2	F 5′‐TAACAGGCCACTGACCATGA‐3′	
	R 5′‐TCTCTTGCATGCACTTGGAG‐3′	
GATA3	F 5′‐CCGAAACCGGAAGATGTCTA‐3′	
	R 5′‐AGATGTGGCTCAGGGATGAC‐3′	
KLF2	F 5′‐CTGCGTACACACACAGGTGA‐3′	
	R 5′‐GTGGCACTGAAAGGGTCTGT‐3′	
18s rRNA	F 5′‐CGCGGTTCTATTTTGTTGGT‐3′	
	R 5′‐AGTCGGCATCGTTTATGGTC‐3′	

F, forward; R, reverse.

### Statistics

The data were expressed as the means ± standard error (SE), and the difference between the averages was assessed using the Mann–Whitney *U*‐test. A *P*‐value of ˂ 0.05 on the basis of at least three independent sets of experiments was considered to be statistically significant.

## Results

### FGF11 is differentially expressed in two phases of adipocyte differentiation

**Figure 1:**
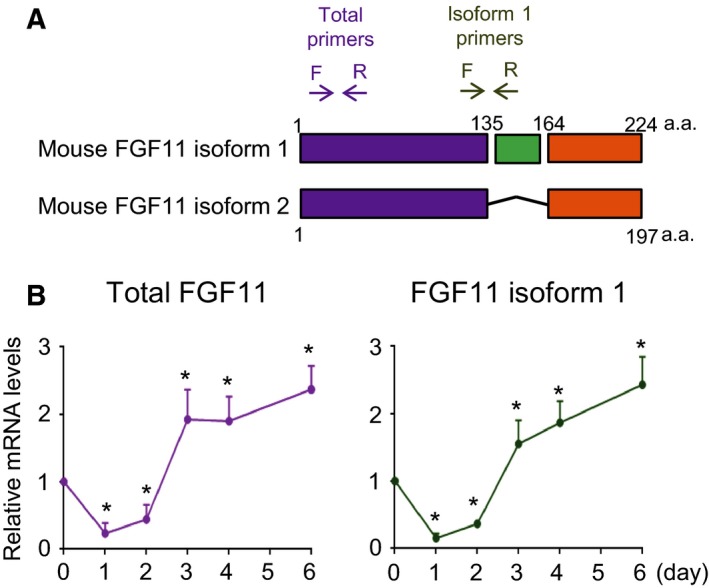
The mRNA level of FGF11 during adipogenesis. (A) Schematic diagram of mouse FGF11 isoforms. The arrows represent the positions of specific primers. Total primers, the position of the specific primers for total FGF11; isoform 1 primers, the position of the specific primers for FGF11 isoform 1. (B) Growth‐arrested 3T3‐L1 preadipocytes were incubated with differentiation inducers, and total mRNA was harvested daily. The FGF11 mRNA level was determined by real‐time RT/PCR and normalized to the 18s rRNA level. The value from harvested cells just before the adipogenic induction (day 0) was set to 1, and the others were calculated relative to this value (mean ± SEM, *n* = 4). The significance was assessed using the Mann–Whitney *U*‐test. *P < 0.05 vs day 0.

The FGF11 of both mouse and human has two isoforms: isoform 1 and isoform 2. Human FGF11 isoform 1 had 97.3% (219/225) sequence identity with mouse FGF11 isoform 1 (Figs 1A and S1). Unlike FGF11 isoform 1, human FGF11 isoform 2 had different features from mouse FGF11 isoform 2. Because the features and sequences of mouse FGF11 isoform 1 showed similarity with human FGF11 isoform 1, we focused on the roles of FGF11 isoform 1 in this study. We measured the expression levels of total FGF11 and FGF11 isoform 1 in 3T3‐L1 using real‐time PCR with specific primers during adipogenesis (Fig. 1B). The expression of total FGF11 and FGF11 isoform 1 showed similar patterns. Interestingly, the expression of FGF11 changed depending on the phase of adipogenesis. Compared with FGF11 levels before the initiation of MCE, FGF11 expression decreased below 20% in the MCE stage (day 0–2), but it then increased approximately twofold in the terminal differentiation stage (day 3–6). The temporal regulation of FGF11 expression during adipogenesis suggests a role for this protein in this process.

### FGF11 knockdown inhibits adipocyte differentiation

**Figure 2:**
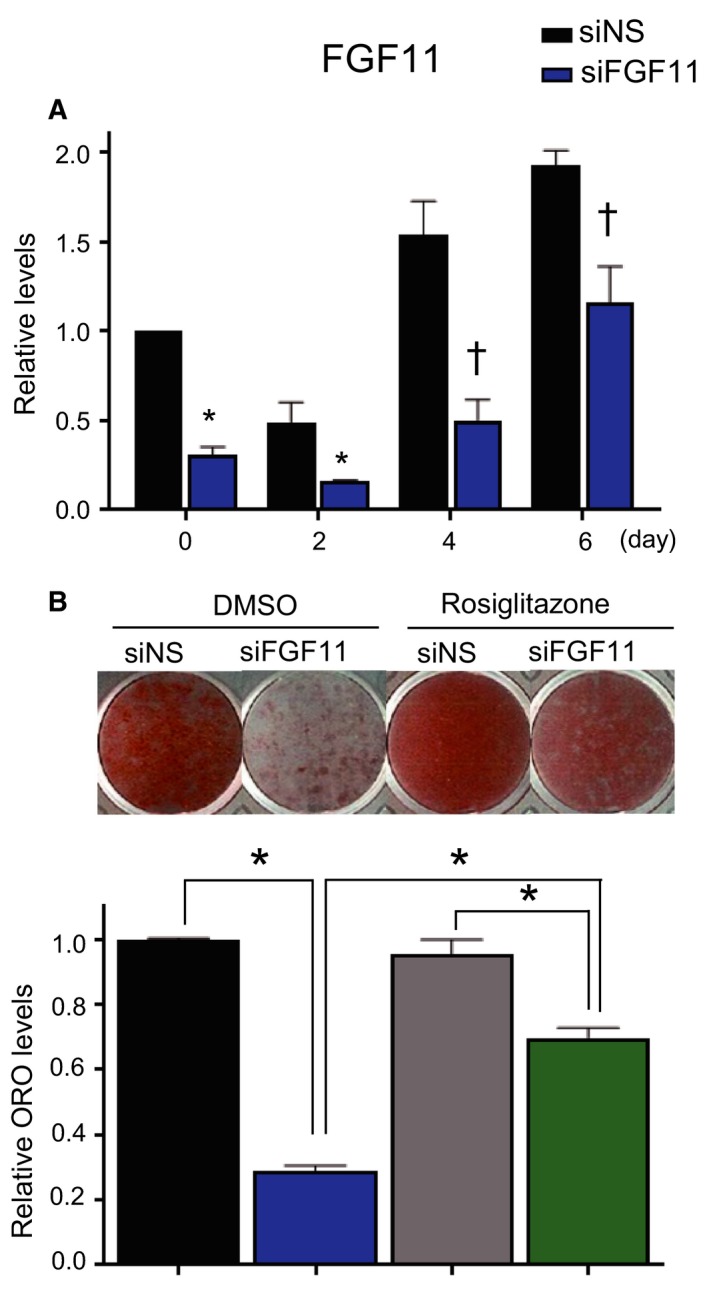
FGF11 knockdown inhibits adipocyte differentiation, and treatment with rosiglitazone significantly alleviates FGF11 inhibitory effect. Growth‐arrested 3T3‐L1 preadipocytes were transfected with siFGF11 or siNS (50 nm) 1 day before adipogenic induction. (A) The transcript levels of FGF11 at the indicated days were determined by real‐time PCR. (*n* = 3, average ± SEM). The significance was assessed using the Mann–Whitney *U*‐test. *P < 0.05 vs siNS at the same time point. †*P* = 0.05 vs siNS at the same time point. (B) Cells were treated with rosiglitazone (10 μm), a PPARγ agonist, from day 2 to 8 of adipogenesis. Cells were stained with ORO at day 8 of adipogenesis (upper panel). The levels of ORO staining were quantified (lower panel). The values of siNS‐ and DMSO‐treated cells were set to 1, and the other values were calculated relative to this value (*n* = 4, average ± SEM). The significance was assessed using the Mann–Whitney *U*‐test. *P < 0.05.

The expression of FGF11 was obviously elevated in the terminal differentiation stage. To investigate whether the increase of FGF11 is involved in adipogenesis, siRNAs against FGF11 isoform 1 (siFGF11) were transfected into 3T3‐L1 preadipocytes 1 day before differentiation induction. Transfection of siFGF11 prevented the elevation of FGF11 and maintained a low level of FGF11 until 6 days after adipogenic stimulation (Fig. 2A). The siFGF11‐transfected cells showed remarkably low amounts of lipid droplets stained by ORO compared to control cells (Fig. 2B). The knockdown of FGF11 accompanied a decrease in adipocyte differentiation, indicating the involvement of FGF11 in adipogenesis.

During adipogenesis, PPARγ is known to be induced beginning at day 2 after adipogenic stimulation, and it plays a pivotal role in terminal adipocyte differentiation 13. To investigate whether PPARγ is involved in the reduced adipocyte differentiation observed in FGF11‐depleted cells, we increased the transcriptional activity of PPARγ by treatment with a PPARγ agonist, rosiglitazone 14 beginning on day 2 after adipogenesis induction. The reduced lipid level induced by FGF11 knockdown was mostly restored by treatment with rosiglitazone, although the restoration was not complete (Fig. 2B), implying that lower PPARγ is associated with the inhibition of adipogenesis by FGF11 knockdown.

### FGF11 knockdown inhibits the expression of PPARγ and C/EBPα

**Figure 3:**
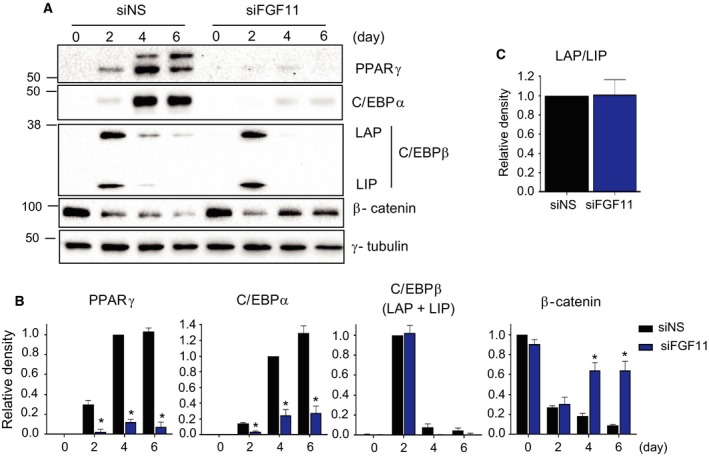
FGF11 knockdown inhibits adipogenesis. One day before differentiation induction, growth‐arrested 3T3‐L1 preadipocytes were transfected with 50 nm of two kinds of specific siRNAs against FGF11 (siFGF11) or nonspecific siRNAs (siNS). (A) Protein samples at the indicated days were subjected to western blotting. (B) The intensity of each band in (A) was normalized to γ‐tubulin. The maximum values of siNS‐treated cells were set to 1, and the other values were calculated relative to this value (*n* = 4, average ± SEM). The significance was assessed using the Mann–Whitney *U*‐test. *P < 0.05 vs siNS at the same time point. (C) The density of C/EBPβ at day 2 in (A) was quantified. The density of C/EBPβ LAP was normalized to that of C/EBPβ LIP. (*n* = 4. average ± SEM).

To investigate how FGF11 affects 3T3‐L1 adipogenesis, we measured the levels of key regulators of adipogenesis in FGF11‐depleted cells and control cells. The expression of PPARγ and C/EBPα, essential regulators of terminal adipocyte differentiation, was induced on day 2 at the end of the MCE phase and was increased during terminal differentiation in cells transfected with siNS (Fig. 3A,B). In contrast, the expression of these proteins was greatly reduced in FGF11‐deficient cells throughout the adipocyte differentiation phases, consistent with Fig. 2. This result demonstrates that FGF11 is required for the induction and maintenance of PPARγ and C/EBPα during adipogenesis.

CCAAT/enhancer‐binding protein β is a positive regulator of the expression of PPARγ and C/EBPα during adipocyte differentiation. The long C/EBPβ isoform [liver‐enriched activating protein (LAP)] is a known transcriptional activator, whereas the truncated isoform LIP, or liver‐enriched inhibitory protein, has been reported to be a dominant‐negative repressor 15. To determine whether FGF11 induces PPARγ and C/EBPα expression by regulating C/EBPβ expression, the levels of C/EBPβ were examined. Total protein levels of C/EBPβ in FGF11‐depleted cells were similar to those of the siNS‐treated sample during the MCE phase (Fig. 3B), and the ratio of LAP/LIP was not changed by FGF11 knockdown (Fig. 3C). These data imply that the expression of C/EBPβ is not associated with FGF11, and the reduced expression of PPARγ and C/EBPα is not due to the failure of C/EBPβ induction during the MCE phase.

β‐Catenin is known to suppress adipogenesis by reducing the expression of PPARγ and C/EBPα 16, 17. To investigate whether the expression of β‐catenin was also influenced by FGF11 expression, the β‐catenin protein levels were monitored during adipogenesis. In control cells, the expression of β‐catenin was significantly decreased from the baseline levels after induction of MCE and was further reduced during terminal differentiation. However, in FGF11‐depleted cells, the expression of β‐catenin was reduced on day 2, similar to the level observed in the control cells, but it increased during terminal differentiation. These results raise a possibility that β‐catenin may be related to the role of FGF11 during terminal differentiation (Fig. 3A,B). However, during adipogenesis, PPARγ downregulated β‐catenin expression by a mechanism that involved proteasomes 18, indicating that the effect of FGF11 knockdown on β‐catenin may be due to an indirect effect of PPARγ cross‐regulation. Taken together, these data suggest that FGF11 knockdown inhibits adipocyte differentiation by regulating the protein levels of the main factors in adipogenesis, such as PPARγ, C/EBPα, and β‐catenin.

### Expression of FGF11 regulates adipogenesis by modulating the expression of PPARγ and C/EBPα

**Figure 4:**
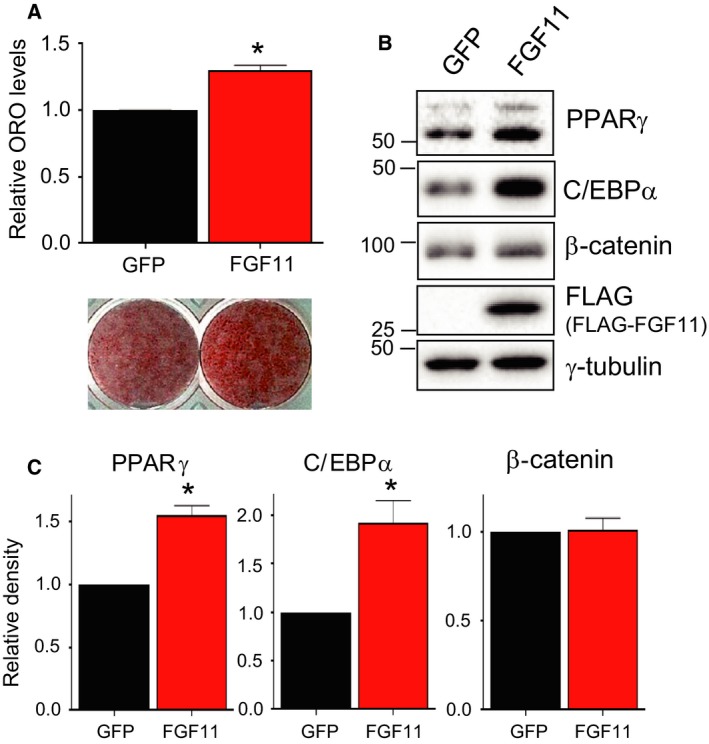
PPARγ and C/EBPα are increased by FGF11 overexpression. Cells were transduced with FLAG‐tagged FGF11‐expressing adenoviruses (FGF11) or control adenoviruses (GFP) at 600 MOI 2 days after the induction of differentiation. (A) Cells were stained with ORO on day 6 after the transduction of the viruses (at day 8 of adipogenesis, lower panel). The stained level was quantified (upper panel) (*n* = 4, average ± SEM). The significance was assessed using the Mann–Whitney *U*‐test. *P < 0.05 vs GFP (B) Differentiation progressed for 2 days after the transduction of viruses, and protein samples were prepared for western blotting. (C) The level of each protein in (B) was quantified. The value from GFP‐treated cells was set to 1, and the other was calculated relative to this value (*n* = 3, average ± SEM). The significance was assessed using the Mann–Whitney *U*‐test. *P < 0.05 vs GFP.

**Figure 5:**
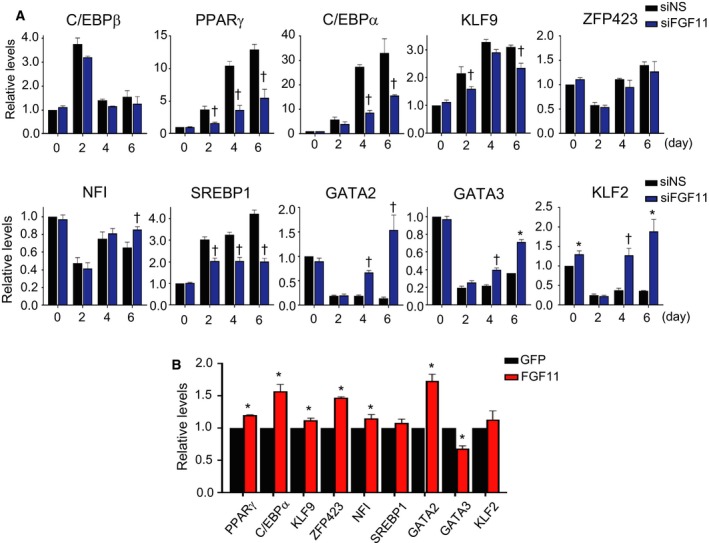
The expression level of genes involved in adipogenesis by FGF11 knockdown or FGF11 overexpression. (A) One day before adipogenic induction, growth‐arrested 3T3‐L1 preadipocytes were transfected with 50 nm of siFGF11 or siNS. Total RNAs at the indicated days were isolated and subjected to real‐time RT/PCR using specific primers as described in Table 1. The values of siNS‐treated cells at day 0 were set to 1, and the other values were calculated relative to this value (*n* = 3, average ± SEM). The significance was assessed using the Mann–Whitney *U*‐test. *P < 0.05 vs siNS at the same time point. †*P* = 0.05 vs siNS at the same time point. (B) FGF11 was overexpressed using an adenoviral expression system (600 MOI) on day 2 of adipogenesis. Adipocyte differentiation progressed until day 4 of adipogenesis, and total RNAs were prepared for real‐time RT/PCR using specific primers. The value of GFP of each was set to 1, and the value of FGF11 of each sample was calculated relative to this value (*n* = 3, average ± SEM). The significance was assessed using the Mann–Whitney *U*‐test. *P < 0.05 vs GFP each.

To confirm the effect of FGF11 on 3T3‐L1 adipogenesis, we investigated whether the overexpression of FGF11 enhanced adipogenesis by upregulating PPARγ and C/EBPα. 3T3‐L1 cells were transduced with FGF11‐expressing adenoviruses at day 2 after adipogenic stimulation to mimic the temporal expression of FGF11 during adipogenesis. As shown in Fig. 1B, FGF11 mRNA decreased during the first 2 days of the MCE phase and then abruptly increased to a higher level than that observed in the preadipocyte state. When the cells were stained with ORO on day 6 after transduction, significantly higher amounts of lipid droplets were found in cells overexpressing FGF11, clearly demonstrating that FGF11 has an essential role in adipocyte differentiation (Fig. 4A). The levels of adipogenic regulators were analyzed at day 4 after adipogenic stimulation, which was 2 days after transduction and is the point at which the expression of PPARγ and C/EBPα reached their maximum levels (Fig. 3A,B). The levels of β‐catenin were not changed in cells overexpressing FGF11 (Fig. 4B,C), suggesting that FGF11 is not directly involved in the suppression of β‐catenin during terminal adipocyte differentiation. We observed increases in the protein and mRNA levels of both PPARγ and C/EBPα upon FGF11 overexpression (Figs 4B,C and 5B). It is known that PPARγ and C/EBPα are cooperatively expressed by a cross‐regulatory mechanism 19, 20. Furthermore, the level of accumulated lipids was also enhanced by FGF11 overexpression from day 2 after adipogenic stimulation (Fig. 4A). Taken together, these results suggest that the regulation of PPARγ expression could be a major target of FGF11 in adipogenesis.

To investigate how FGF11 affects adipogenesis, we assessed the transcript levels of regulatory genes involved in adipocyte differentiation. Among the regulatory genes, we examined the expression of regulators of PPARγ, because PPARγ is likely to be the major mediator of the effects of FGF11 on adipocyte differentiation. As positive regulators of PPARγ 21, the transcripts of KLF9, sterol regulatory element‐binding protein 1 (SREBP1), and C/EBPα were reduced by FGF11 knockdown (Fig. 5A). However, the expression levels of GATA binding factor 2 (GATA2), GATA binding factor 3 (GATA3), and KLF2, which are negative regulators of PPARγ 21, were upregulated by FGF11 knockdown (Fig. 5A). When FGF11 was overexpressed in 3T3‐L1 cells beginning on day 2 of adipogenesis, the expression levels of KLF9, zinc finger protein 423 (ZFP423), nuclear factor I (NFI), and C/EBPα, which are positive regulators of PPARγ, were increased, whereas GATA3 transcripts were decreased (Fig. 5B). Contrary to the behavior of the other negative regulators, GATA2 expression was increased by FGF11 overexpression (Fig. 5B). These data could be partly explained by the results of a previous study, in which GATA2 negatively regulates the expression of PPARγ in the early stage of adipogenesis, whereas the relationship between GATA2 and PPARγ is changed in the mature adipocytes 22. The transcript analysis of PPARγ regulators suggests that FGF11 expression may modulate PPARγ transcription by changing the expression of PPARγ regulators such as C/EBPα, KLF9, SREBP1, GATA2, GATA3, and KLF2.

**Figure 6:**
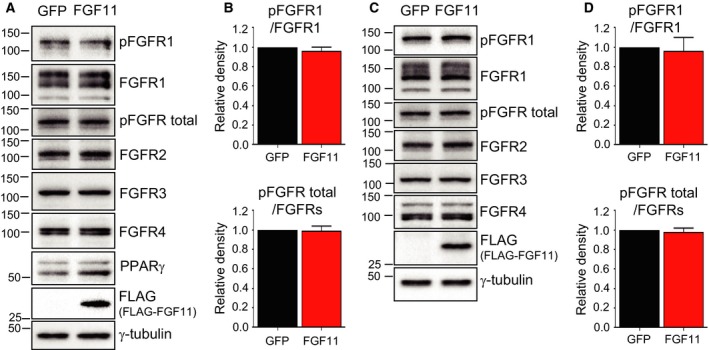
FGF11 overexpression did not affect the levels of phosphorylation of FGFRs. (A) FLAG‐tagged FGF11 was overexpressed by adenoviral expressing system (600 MOI) from day 2 to 4 of adipogenesis. Protein samples were prepared for western blot. (B) The density of phosphorylated FGFR1 (pFGFR1)/FGFR1 or pFGFR total/total FGFRs in (A) was quantified. The level from GFP‐treated cells was set to 1, and the other was calculated relative to this value (lower panel) (*n* = 3, average ± SD). (C) Fully differentiated cells were transduced with 600 MOI of FLAG‐tagged FGF11 expressing adenoviruses (FGF11) or control adenoviruses. After 2 days, protein samples were prepared and subjected to western blot. (D) The density of pFGFR1/FGFR1 or pFGFR total/FGFRs in (C) was quantified. The level from GFP‐treated cells was set to 1, and the other was calculated relative to this value (lower panel) (*n* = 3, average ± SD).

To investigate whether intracrine FGF11 activates FGFR during 3T3‐L1 preadipocyte differentiation like other secretory FGFs, the expression and phosphorylation of FGFRs in FLAG‐FGF11‐overexpressed 3T3‐L1 cells were monitored. When FLAG‐tagged FGF11 was overexpressed by adenoviral expressing system from day 2 to 4 of adipogenesis and in fully differentiated cells, the expression of FGFRs (1–4) and the levels of phosphorylated FGFR 1 and total FGFR were not changed (Fig. 6), which indicates the regulatory mechanism of adipogenesis by FGF11 is different from that of other FGFs.

## Discussion

Fibroblast growth factors function as important factors in a variety of biological processes such as embryonic development, tissue maintenance, repair, regeneration, survival, and metabolic regulation. FGF11 belongs to iFGFs group, which show different biochemical and functional characteristics than the typical FGF group. While other iFGFs have received much attention for their roles and molecular mechanisms, FGF11 has not been well studied. However, recent research has revealed novel characteristics of FGF11 that other iFGFs do not share. Here, we found that FGF11 expression exerts a regulatory effect on the adipogenesis of 3T3‐L1 cells. This novel function of FGF11 on preadipocyte differentiation was not disclosed in other intracrine FGFs, even though other endocrine or paracrine/autocrine FGFs are implicated in the adipogenesis. FGF1 was suggested as a key human adipogenic factor 23 and has emerged as a potential drug candidate for the treatment of type 2 diabetes mellitus 24, 25. FGF10 is secreted by cultured preadipocytes and plays an important role in adipogenesis in a paracrine or autocrine manner 26 by activating FGFR2b with heparin/heparin sulfate as a cofactor 27. Endocrine FGFs such as FGF19 and FGF21 act as important metabolic regulators 28. The incubation of 3T3‐L1 with FGF21 stimulates the phosphorylation of signaling molecules and preadipocyte differentiation 29, and FGF21 requires βklotho to form the FGF21‐FGFR complex and modulate glucose uptake in 3T3‐L1 cells 30. Since FGF21 has different characteristics from other metabolic regulators, FGF21 gains attention as an agent to treat metabolic disease 31. To date, most of the research regarding the relationship between FGF and adipogenesis has focused on secretory FGFs. We suggest that endogenous FGF of the intracrine FGF11 can regulate FGFR‐independent preadipocyte differentiation (Fig. 6), which is a different regulatory mechanism in adipogenesis from other FGFs.

Fibroblast growth factor 11 has an N‐terminal nuclear localization sequence (NLS) 32, but it has been reported that FGF11 is localized in the cytosol as well as the nucleus 5, 33. Therefore, the presence of the NLS in FGF11 isoforms could have a profound impact on their localization and function. Mouse FGF11 isoform 2 does not have the 27 amino acid sequence in the middle region, compared to the two human FGF11 isoforms and mouse FGF11 isoform 2, and the remaining sequences are quite similar. In contrast, only human FGF11 isoform 2 lacks an N‐terminal NLS. Unlike FGF11 isoform 2, human FGF11 isoform 1 and mouse FGF11 isoform 1 share high similarity (Fig. S1). Due to these distinct features of FGF11 isoform 2, the roles of the mouse FGF11 isoform 2 might be different from those of the human FGF11 isoform 2. Hence, FGF11 isoform 1 was preferentially selected as the FGF11 isoform for this study.

**Figure 7:**
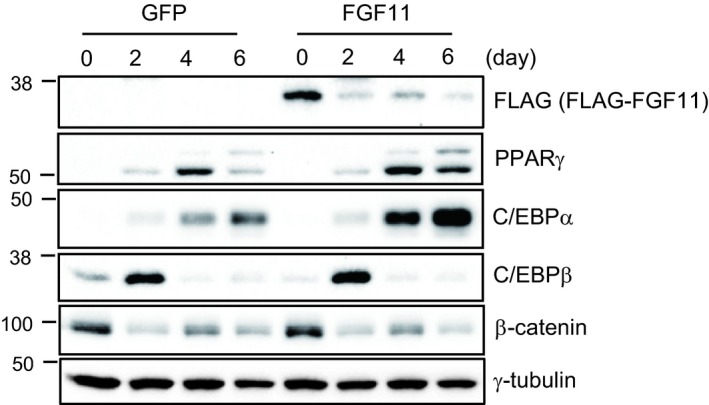
Exogenous FGF11 was dramatically decreased in the MCE phase. Growth‐arrested 3T3‐L1 cells were transduced with FLAG‐FGF11 expressing adenoviruses at 1 day before adipogenic stimulation, subsequently induced adipogenesis. Protein samples at the indicated days were subjected to western blot.

First, we observed dynamic changes in the expression of FGF11 during adipogenesis. In the MCE phase, FGF11 expression remained at low levels (day 0–2). In the terminal differentiation phase (day 3–6), however, FGF11 expression dramatically increased to approximately twofold higher than the level observed before adipogenic stimulus. To explore whether reduction of FGF11 in the MCE phase is involved in adipogenesis, we overexpressed FGF11 in 3T3‐L1 preadipocytes under the MCE phase. Growth‐arrested cells were transduced with FGF11‐expressing adenoviruses 1 day before differentiation induction, and subsequently, the process of adipocyte differentiation was initiated. Surprisingly, the protein level of exogenous FLAG‐tagged FGF11 was extremely low after the stimulation of adipogenesis, even though the protein was overexpressed well at day 0 (Fig. 7). Since FGF11 was overexpressed under the control of the CMV promoter, FGF11 could be regulated post‐transcriptionally. The decrease of endogenous FGF11 just after MCE initiation (Fig. 1B) could be forced by a regulatory mechanism. This result raised the possibility that the reduction in FGF11 in the MCE phase is an important regulatory process during adipogenesis. Further studies will be performed to determine why FGF11 expression is maintained at a low level. When the increase of FGF11 was blocked by FGF11 knockdown in the terminal differentiation phase, the induction of PPARγ and C/EBPα, well‐known critical adipogenic regulators, was mostly attenuated, resulting in the inhibition of adipogenesis. In contrast, the expression of PPARγ and C/EBPα was increased and adipocyte differentiation was enhanced by FGF11 overexpression in the terminal differentiation phase. These results indicate the importance of the increase in FGF11 during the terminal differentiation phase. Taken together, the data suggest that the expression of FGF11 may be a regulatory component involved throughout the entire process of adipogenesis.

**Figure 8:**
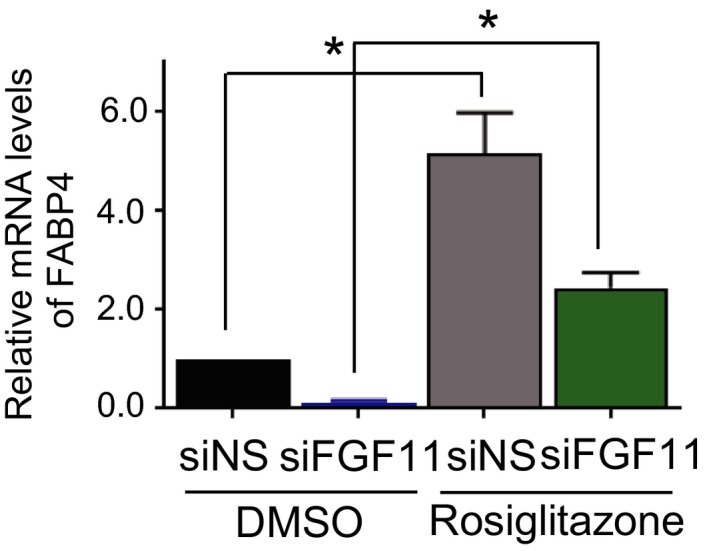
Expression of FABP4 was decreased by FGF11 knockdown. Second day after treatment of rosiglitazone (at day 4 of adipogenesis), total RNAs were isolated and subjected to real‐time RT/PCR. The value of siNS and DMSO‐treated cells was set to 1, and the other values were relatively calculated (*n* = 3, average ± SEM). The significance was assessed using the Mann–Whitney *U*‐test. *P < 0.05.

Peroxisome proliferator‐activated receptor gamma is a major regulator of adipocyte differentiation. Since the expression level of PPARγ was lower in FGF11‐depleted cells than in control cells, we increased the transcriptional activity of PPARγ by treatment with a PPARγ agonist, rosiglitazone, to determine the involvement of PPARγ in the FGF11 effect. The inhibition of adipocyte differentiation by FGF11 knockdown was significantly restored by the addition of rosiglitazone. These results suggest that a low level of PPARγ may be a necessary factor for the attenuated adipogenesis induced by FGF11 knockdown. However, adipocyte differentiation was partly recovered. It is likely that the remaining PPARγ level is too low for differentiation to be fully recovered. This result coincides with the expression of FABP4 (Fig. 8), which is downstream of PPARγ and regulates lipid accumulation 34.

Peroxisome proliferator‐activated receptor gamma expression is controlled by multiple positive and negative transcription factors during adipogenesis 21. Here, we examined the expression levels of several regulators of PPARγ expression: C/EBPβ, C/EBPα, KLF2/9, ZFP423, NFI, SREBP1, and GATA2/3. We found that several regulators were involved in PPARγ expression based on the transcriptional changes of regulators by FGF11 knockdown or overexpression: C/EBPα, KLF9, SREBP1, GATA2, GATA3, and KLF2. The transcript levels of negative regulators of PPARγ expression, including GATA2, GATA3, and KLF2, were obviously altered by the level of FGF11 compared to those of positive regulators (Fig. 5), although the level of GATA2 exhibited an unexpected increase in FGF11‐overexpressed cells. Any single gene among GATA2, GATA3 35, KLF2 36, and SREBP1 37 can exert PPARγ‐dependent regulatory effects on adipogenesis, which means that the FGF11 effect on adipogenesis is attributed to the combined action of these PPARγ regulators controlled by FGF11. FGF11 expression could modulate the expression of each PPARγ regulator or control a common factor upstream of the PPARγ regulators.

In summary, we hypothesized that the expression of FGF11 is regulated precisely during the MCE phase and the terminal differentiation phase. We showed a decrease of PPARγ by FGF11 knockdown, an increase of PPARγ by FGF11 overexpression, and a recovery of adipogenesis by treatment with a PPARγ agonist. These results suggest that the expression of PPARγ is a major target of FGF11 during adipogenesis, and the precise regulation of the expression of FGF11 may play an important role in adipogenesis. In addition, we demonstrated that the expression of C/EBPα, KLF9, SREBP1, GATA2, GATA3, and KLF2 was influenced by FGF11 expression, which results in the regulation of PPARγ. In conclusion, we suggest that FGF11 is a novel mediator of adipogenesis and indirectly controls the expression of PPARγ by modifying the expression of regulators of PPARγ expression. The effect of FGF11 on adipogenesis may be an integrated outcome through the expression of many PPARγ regulators that are controlled by FGF11.

## Conflict of interest

The authors declare no conflict of interest.

## Author contributions

JHL and HSY conceived and supervised the study; KWL, HSY, and JHL designed the experiments; KWL and YJA performed the experiments; KWL, HSY, and JYJ analyzed the data; and KWL, JYJ, and HSY wrote the manuscript.

## Supporting information


**Fig. S1.** Multiple amino acid sequence alignment of mouse and human FGF11 isoforms.
