# Supplementary material for: FGF11 influences 3T3‐L1 preadipocyte differentiation by modulating the expression of PPARγ regulators
Source: FEBS Open Bio. 2019 Mar 12;9(4):769–80. doi: 10.1002/2211-5463.12619 (PMC6443871; doi:10.1002/2211-5463.12619)
Supplement: Supplementary file 1 — Fig. S1. Multiple amino acid sequence alignment of mouse and human FGF11 isoforms. [file FEB4-9-769-s001.pptx]

## Slide 1
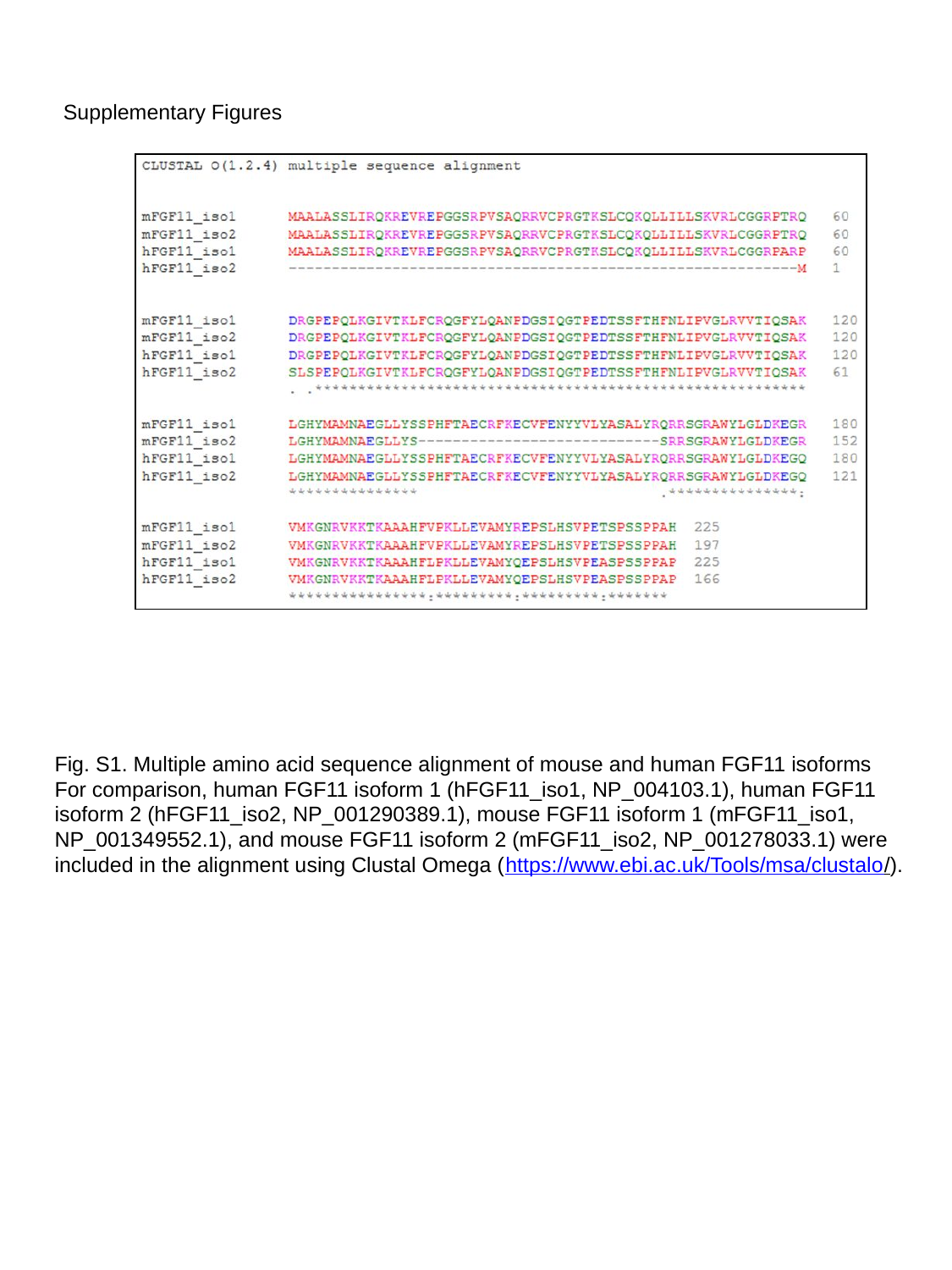

Supplementary Figures
Fig. S1. Multiple amino acid sequence alignment of mouse and human FGF11 isoforms
For comparison, human FGF11 isoform 1 (hFGF11_iso1, NP_004103.1), human FGF11 isoform 2 (hFGF11_iso2, NP_001290389.1), mouse FGF11 isoform 1 (mFGF11_iso1, NP_001349552.1), and mouse FGF11 isoform 2 (mFGF11_iso2, NP_001278033.1) were included in the alignment using Clustal Omega (https://www.ebi.ac.uk/Tools/msa/clustalo/).
